# Vagal Paraganglioma: A Rare Finding in a 31-Year-Old Male

**DOI:** 10.7759/cureus.18423

**Published:** 2021-10-01

**Authors:** Yashfeen Ahmed, Anum Arif, Ahsin Manzoor Bhatti, Shahbaz Ali Nasir, Sabih Nofal, Ali Hamza, Usman Jamil Mughal

**Affiliations:** 1 Internal Medicine, Combined Military Hospital Lahore, CMH Lahore Medical College, Lahore, PAK; 2 Vascular Surgery, Combined Military Hospital Lahore, CMH Lahore Medical College, Lahore, PAK; 3 Vascular surgery, Combined Military Hospital Rawalpindi, Rawalpindi, PAK

**Keywords:** key words: carotid body tumor, fever in vagus nerve paraganglioma, paraganglioma, head and neck neoplasms, vagus nerve, glomus vagale, general and vascular surgery, vagal paraganglioma

## Abstract

Vagal paraganglioma is a rare finding that develops from paraganglionic tissue found around the vagus nerve; it has a prevalence of 0.012% of all tumors. It is the third most common paraganglioma of the head and neck but still accounts for less than 5% of these tumors, and it has a well-established female prevalence. It is a difficult tumor to identify early based on its symptoms alone and only a thorough investigation can help solidify its diagnosis. In this report, we discuss a presentation of this phenomenon that is not only unique in its manifestation but also a very difficult diagnosis due to its deceptive presentation and multiple extensions. These masses need a good surgical regime to be removed properly and postoperative complications are very frequent in most of these cases.

## Introduction

Vagal paragangliomas are rare findings that develop from paraganglionic tissue found around the vagus nerve [1]. They are derived from neural crest cells found at or below the inferior ganglion vagus nerve, at the level of the carotid bifurcation, or along the vagus nerve in the mediastinum [2]. The most common paragangliomas of the head and neck include the carotid body tumor, followed by jugulotympanic paragangliomas, and thirdly vagus nerve tumors. Overall, vagal paragangliomas account for less than 5% of all head and neck paragangliomas [1,3]. There is a well-established female predominance with a female-to-male ratio ranging from 2.7:1 to 6:1 [4]. These tumors may be sporadic or familial in variety, with the familial type being autosomal-dominant in inheritance [4]. The most common symptoms arise from the compression of its adjacent structures, and only 5% secrete catecholamines like the paragangliomas of the adrenal gland also known as pheochromocytomas [5]. Paragangliomas, in general, account for 0.012% of all tumors and only 10% of them have extra-adrenal localization. Head and neck paragangliomas are even rarer with an incidence of 0.33%. Typically, paragangliomas are benign tumors, but around 19% of cases may have malignant potential. Vagal paragangliomas often present a surgical challenge due to their proximity to the internal and external carotid arteries, the lower cranial nerves, the internal jugular vein, and their propensity to extend to the skull base [6,7]. They have an annual incidence of one per 100,000 population. There is an obvious female predominance and the mean reported age at diagnosis is 45 years [8]. In this report, we present a case of a 31-year-old male with a rare vagal nerve paraganglioma.

## Case presentation

A 31-year-old male presented to the vascular surgery clinic with a history of multiple neck swellings and intermittent fever for six years. Initially, it had been just one swelling on the right lateral neck but over time, the swellings had increased in size and number. They were painful and tender to the touch and the pain intensity had increased over the years. The pain was non-radiating and was not associated with any nausea or vomiting, no difficulty in breathing, or weight loss. However, the swellings were associated with a fever of 101-102 °F. The fever was intermittent in pattern, was not associated with any rigors and chills, any cough, or sore throat. The fever was relieved upon taking acetaminophen, and the patient's workup for other causes responsible for the fever was unremarkable.

The patient had woken up one day with a stiff neck and had been unable to rotate his neck with only slight movements possible. He had experienced excruciating pain in the neck swelling, which he described as an 8/10 in intensity. This pain he described had radiated from his throat into his right ear. He had also complained of pain in his right shoulder but no loss of shoulder movement. He had also started experiencing pain in the ipsilateral ear along with tinnitus. Along with the pain, he had experienced dysphagia. There had been no loss of hearing, no fainting spells, or hoarseness of voice. This had been accompanied by a loss of control of his right lower lip.

**Figure 1:**
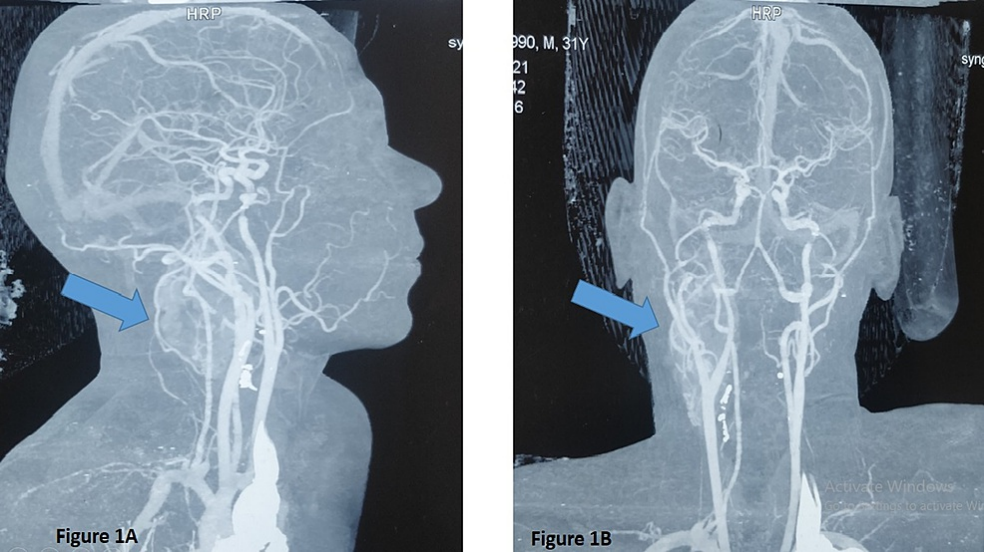
MRI with contrast sagittal view (Figure 1A) and craniocaudal view (Figure 1B) Blue arrow indicates a mass of 50 x 26 x 28 mm on the right side of the neck causing splaying of the internal carotid artery and external carotid artery MRI: magnetic resonance imaging

For this episode of neck stiffness, he had gone to the local general practitioner where an ultrasound of his neck had been performed, which had revealed a right carotid body tumor. The general practitioner had prescribed pain medications and referred him to the hospital. He had been referred from one hospital to the other until he had reached a tertiary care hospital where an MRI with contrast of the neck had revealed a mass, measuring 50 x 26 x 28 mm on the right side of the neck extending superiorly up into the base of the skull and inferiorly at the level of the thyroid cartilage, as shown in Figure 1A and Figure 1B. The mass had been posterior to the right carotid bifurcation, external carotid artery, and internal carotid artery. There had been anterior splaying of both the internal and external carotid arteries. Superiorly, the internal carotid artery had been completely encased; while inferiorly, it had been partially encased. These findings had further strengthened the diagnosis of a carotid body tumor.

The patient was referred to the vascular surgery department of our institution in June 2021. His case was discussed in a multidisciplinary committee with the radiologists, which recommended surgical excision.

**Figure 2:**
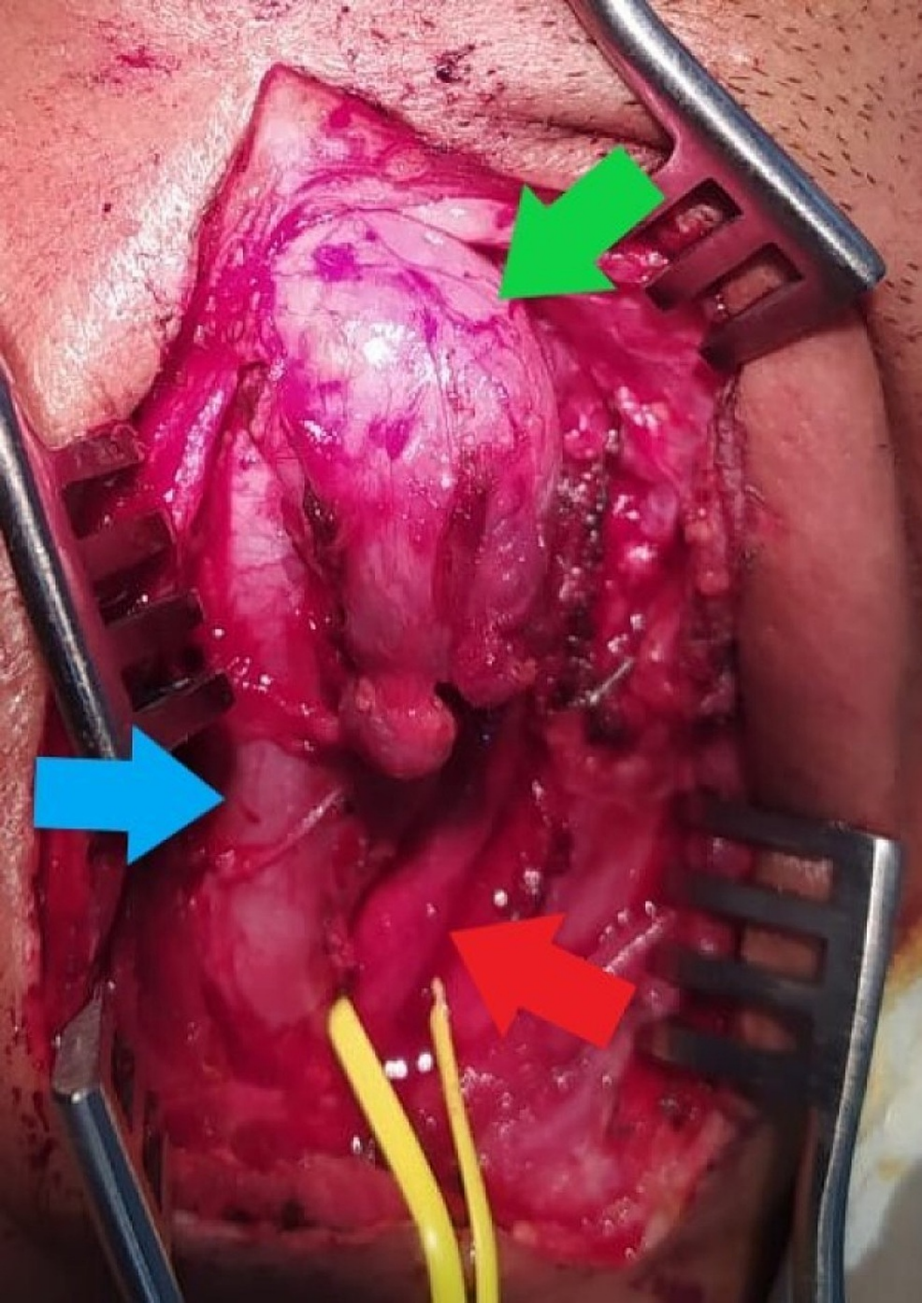
Intraoperative findings - image 1 The green arrow indicates the globular appearance of the tumor. The red arrow shows the common carotid artery (CCA). The blue arrow indicates the internal jugular vein (IJV). Note the splaying of the IJV and CCA by the tumor

**Figure 3:**
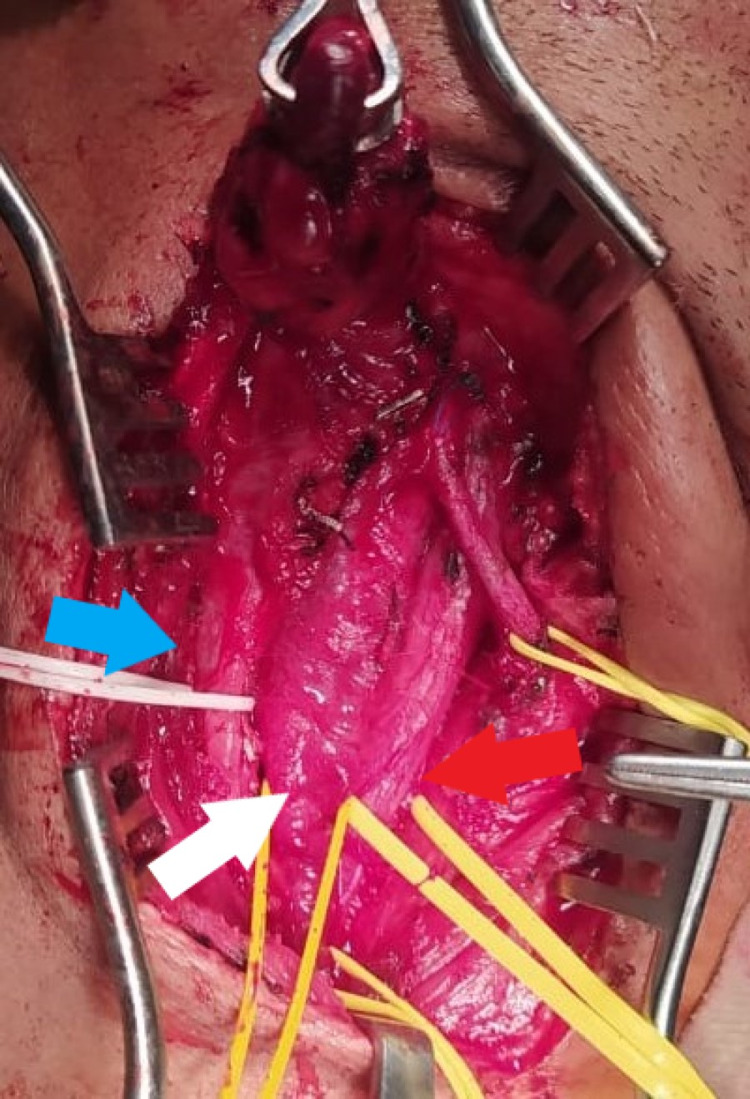
Intraoperative findings - image 2 The green arrow indicates the lifted globular part of the tumor. The red arrow indicates the common carotid artery (CCA). The blue arrow indicates the internal jugular vein (IJV). The white arrow indicates the part of the tumor in between the CCA and IJV causing them to deviate apart. Note that no carotid bifurcation is visible in the neck upto the angle of mandible, indicating high carotid bifurcation

Under general anesthesia, an incision was made in front of the anterior border of the sternocleidomastoid muscle, extending to the tragus. We noticed a globular, cystic, non-pulsatile mass adherent to the carotid sheath. Upon opening the sheath, the mass had ramifications between the common carotid artery and internal jugular vein, reaching up into sphenoid recess superiorly and inferiorly; the tumor was found encasing the vagus nerve, as shown in Figure 2. There was a high carotid bifurcation at the level of mandible angle, which was partially encased by the tumor, as shown in Figure 3. The vagus nerve was excised along with the tumor and the internal jugular vein was double-ligated, as it was found to be already thrombosed. The tumor was separated from the encased internal carotid artery with careful dissection. The tumor was close to the 12th cranial nerve, which was fixed by the tumor. The facial nerve could not be identified separately from the tumor. The tumor was carefully excised and the patient was then shifted to the surgical intensive care unit.

On the immediate postoperative day, the patient developed hoarseness, difficulty in swallowing, and deviation of the angle of the mouth to left. This was attributed to the marginal mandibular nerve being injured during the operation, despite careful dissection. He also lost his ipsilateral nasolabial folds. He was put on nasogastric feeding, which was switched to semisolid food on the third postoperative day. His hoarseness settled on the second postoperative day. On indirect laryngoscopy, ipsilateral vocal cord paralysis was confirmed. His hoarseness was temporary as the damaged vocal cord was compensated for by the adjacent vocal cord. He was discharged home on the fourth postoperative day on a semisolid oral diet. The histopathological report confirmed the suspicion of vagus nerve paraganglioma and indicated an encapsulated tumor with zellballen architecture. The sample stained positive for S100, synaptophysin, and chromogranin. The lymph nodes examined showed no evidence of tuberculosis or malignancy. In conclusion, a paraganglioma of the vagus nerve with reactive hyperplasia of the neck nodes was present.

## Discussion

Vagal paragangliomas usually arise in the parapharyngeal space though there is evidence of cervical paragangliomas growing along the cervical course of the vagus nerve [8]. They frequently present as rubbery neck masses, may transmit the carotid pulse if it enlarges and encircles around the carotid artery, but they can also cause tinnitus, dysphagia, hoarseness, horners syndrome (if the cervical sympathetic chain is invaded), or other cranial nerve deficits depending upon their site of involvement [5,6,9,10]. These patients present with cranial nerve palsies in about 50% of cases [11]. In our case, the patient also experienced similar symptoms of a stiff neck, dysphagia, and tinnitus along with lower facial nerve palsy symptoms with sparing of the superior functions of the right facial nerve. No relationship has been documented in the available literature relating fever with a vagus nerve paraganglioma; however, in our case, a direct relationship was found, as the patient's fever improved postoperatively.

The association of head and neck paragangliomas with catecholamine hypersecretion is a rare incidence, and hence, urinary catecholamine screening is often performed only in the presence of symptoms like tachycardia, hypertension, or, as in our case, a family history of paraganglioma [6]. Just like many others, our patient had no elevated blood pressure, headache, tachycardia, tremors, shortness of breath, or any other catecholamine hypersecretion features.

Genetic tests are recommended in patients with any of the following risk factors: >50 years of age, family history, extra-adrenal tumors, multiple and/or metastatic tumors, and/or elevated dopamine and methoxytyramine levels. In the patient presented in this report, genetic testing was not performed owing to a negative personal, medical, and family history of paragangliomas or other neuroendocrine tumors [5].

Preoperative diagnosis of vagal paraganglioma, even though difficult, can be made by combining clinical findings and radiographic studies [11]. These tumors are easily diagnosed by MRI and, in some cases, with the addition of MR angiography. Contrast-enhanced CT and MRI depict these highly vascular, soft tissue masses equally well. Specific imaging characteristics, like salt-and-pepper appearance due to flow voids in contrast to MRI, are crucial to differentiate carotid body paragangliomas from other tumors of the parapharyngeal space. CT is indicated for tumors invading the skull base to better delineate the details of the bony erosion and the extent of involvement. MRI is indicated in most cases and is complementary to CT. It will be evident radiologically that vagal paragangliomas are located behind the internal carotid artery, unlike carotid body paragangliomas, which are found at the carotid bifurcation. Vagal paragangliomas characteristically displace both the internal and external carotid arteries anteriorly, while the internal jugular vein is compressed and displaced posteriorly [5,6,8,9,12]. Our patient also underwent similar investigations and a mass was found posterior to the right carotid bifurcation, external carotid artery, and internal carotid artery. Splaying of the internal carotid and external carotid arteries along with its anterior displacement was found. However, the presentation of this tumor was rather deceptive, as it was a relatively large tumor with multiple extensions that included the internal carotid artery completely superiorly and partially inferiorly, which made the exact diagnosis tricky, as shown in Figure 3. Finally, scintigraphy with octreotide and PET-CT can be useful devices to aid in identifying multicentric locations, as these tumors express large numbers of somatostatin receptors [6,8]. However, due to a lack of somatic symptoms, this was also not performed.

The differential diagnosis in our patient involved vagal paraganglioma, schwannoma, neuroma, jugular meningioma, carotid body paraganglioma as well as jugular and tympanic paragangliomas. Due to their similar clinical presentation, they were high in our differentials. However, schwannoma shows moderate enhancement while paragangliomas reveal avid enhancement on imaging. Histologic characteristics of schwannomas include alternating Antoni A and B regions, which are distinctly different from paragangliomas. Displacement of the common or internal carotid arteries is also characteristic of these tumors. They do not demonstrate flow voids [5,13]. Hence our initial differentials also revolved around this list and investigations, an anatomical position once exposed intraoperatively, and postoperative histological confirmation.

Vagus paraganglia are morphologically identical to the carotid body with glomus cells (type I), located within the nerve or adjacent to the nerve. Both show the characteristic “zellballen” pattern, which is a reticular network of interlacing neuroepithelial sheets of varying thicknesses, separated by a rich microvascular bed. Microscopically, this “zellballen” pattern takes the appearance of roundish or elongated oval clusters of cells [6,14]. The sample we obtained after excision demonstrated this exact pattern.

Vagal paraganglioma in the parapharyngeal space has a rich blood supply and is closely related to the internal and external carotid arteries, internal jugular vein, and posterior cranial nerves. Surgical resection should be initially considered. The key to a successful outcome is a calculated approach to obtain excision of the tumor while paying full attention to protect the internal carotid artery. It can often be difficult to separate the nerve from the tumor capsule [11]. Surgical resection is a primary treatment for vagus paragangliomas, whereas radiation therapy is used in the case of malignant and unresectable tumors. Vagus paragangliomas have a lower risk for metastasis compared with carotid paragangliomas [6].

Surgical morbidity associated with vagal deficits is unavoidable. The literature clearly shows that vagal function cannot always be preserved even when the nerve is anatomically intact [8]. Reports in the literature state that nerve-sparing can only be achieved in 5-8% of patients, including some early cases in which the nerve fibers were splayed over the tumor and other cases in which nerve continuity was preserved by leaving a portion of the tumor adhering to the nerve [11]. Hence cranial nerve dysfunction is a common complication after surgery for cervical paragangliomas and has been reported to occur almost always after the excision of tumors originating from the vagus nerve [12]. A similar outcome was achieved in our case where the patient developed transient hoarseness, dysphagia, and deviation of the angle of the mouth to the left. The hoarseness was present as the vagus nerve was excised but it was transient as the ipsilateral vocal cord provided compensation of function and the hypoglossal nerve was unaffected as the tongue remained central. The deviation of the angle of the mouth was due to damage to the marginal mandibular nerve during the procedure. Dysphagia occurred because of the damage to the ninth cranial nerve during the procedure despite careful dissection. This was the outcome even after extremely careful dissection of the tumor, which is in line with the available literature.

## Conclusions

Paraganglioma of the head and neck is a rare finding, especially in such an age as our patient's. Clinically, it is extremely difficult to differentiate between swellings of the neck as most of them have a similar presentation. CT and MRI are the imaging modalities of choice. Despite performing preoperative radiographic studies (contrast-enhanced CT and digital angiography), the vagal origin of the tumor was a diagnostic surprise. Surgical exploration was performed based on clinical suspicion of a carotid body tumor. Angiographic signs and findings were not accurately analyzed due to the fact that the swelling was behind the carotid bifurcation rather than involving it. Moreover, histologically it is very difficult to differentiate between carotid body paraganglioma and a vagus nerve paraganglioma, as they both have positive stains due to their similar embryological origin. Only the actual position of the swelling can reveal the exact nature of involvement. Histology is extremely useful in deciding if the swelling is metastatic or not. These masses need a good surgical regime to be removed properly and postoperative complications are unavoidable in most cases. However, with good surgical acumen and technique along with a routine follow-up, the prognosis can be easily improved.
